# Editorial

**Published:** 1866-09

**Authors:** 


### Editorial.

CHICAGO MEDICAL SOCIETY.—The regular meeting of this Society was held on the evening of the first Friday in August. Dr. E. L. HOLMES reported a case, in which he had occasion to have a strong solution of atropine dropped into the eye of one of his patients, at short intervals, for twenty-four hours in succession, when severe and well-marked symptoms of the general effect of atropine upon the whole system began to be manifested, and were continued for a considerable time. Dr. NELSON related a case, in which a solution of atropine dropped into the eye twice, caused a dilatation of the pupil that continued through a period of ten days, but was not accompanied by any general disturbance of the system. These cases elicited additional observations and facts from Drs. INGALLS, ROSS, and DAVIS.

*Encephaloid Tumor.*—Dr. BOGUE presented to the Society a tumor taken from the arm of a young girl in Cook County Hospital. The tumor had first made its appearance after a blow on the forearm, one or two years previous. It was about the size of a small hen's egg, and seemed to originate wholly from the skin. It was soft, somewhat lobulated, nearly destitute of fibrous tissue, and a portion of it examined by Dr. LYMAN, presented in the field of the microscope but little else than granules.

*Narcotism.*—Dr. WANZER related a case of poisoning by opium. The patient, a married woman, was represented as having taken two ounces of laudanum, for the purpose of self-destruction. The narcotism was such that the stomach appeared, at first, insensible to the action of an emetic, but dashings of cold water so far aroused the sensibility that free vomiting ensued; after which, the frequent dashing of cold water was kept up for several hours, and the patient recovered.

After some further discussion, the Society adjourned until the third Friday in August.

---

MORTALITY FOR THE MONTH OF MAY.—The mortality report for the month of May, as compiled by the health-officer, is given below. The statistics show an increase of 25 deaths over the corresponding period of last year, the increase being chiefly in those diseases resulting from colds. Consumption, as usual, has reckoned among its victims a large proportion of those who have died. There is no epidemic in the city, and no cases of small-pox have appeared for several months.

CAUSES OF DEATH.

| | | | |
|---|---|---|---|
| Accidents, | 5 | Fever, Typhoid, | 12 |
| Bright's Disease, | 2 | Fever, Typhus, | 1 |
| Bronchitis, | 1 | Hemorrhage, | 1 |
| Cancer, | 3 | Hydrocephalus, | 4 |
| Childbed, | 3 | Inanition, | 1 |
| Cholera Infantum, | 1 | Inflammation, | 2 |
| Colic, | 2 | Inflammation of Brain, | 4 |
| Congestion of Brain, | 6 | Inflammation of Bowels, | 7 |
| Congestion of Lungs, | 3 | Inflammation of Bladder, | 1 |
| Colds, | 2 | Inflammation of Lungs, | 14 |
| Consumption, | 38 | Inflammation of Pleura, | 1 |
| Convulsions, | 23 | Inflammation of Kidneys, | 1 |
| Croup, | 9 | Marasmus, | 1 |
| Diarrhœa, Chronic, | 2 | Measles, | 7 |
| Diphtheria, | 6 | Old Age, | 6 |
| Disease of Bowels, | 1 | Poisoning, | 1 |
| Disease of Bladder, | 1 | Rheumatism, | 2 |
| Disease of Heart, | 5 | Rupture, | 1 |
| Disease of Liver, | 3 | Small-Pox, | 1 |
| Disease of Lungs, | 2 | Stillborn, | 10 |
| Dropsy, | 7 | Tumor, | 1 |
| Drowned, | 4 | Teething, | 14 |
| Dysentery, | 3 | Tuberculosis, | 1 |
| Erysipelas, | 4 | Unknown, | 31 |
| Fever, Childbed, | 2 | Whooping-Cough, | 3 |
| Fever, Congestive, | 2 | | |
| Fever, Scarlet, | 7 | Total, | 275 |

AGES OF THE DECEASED.—Under 5 years, 134; over 5 and under 10 years, 14; over 10 and under 20, 11; over 20 and under 30, 27; over 30 and under 40, 31; over 40 and under 50, 22; over 50 and under 60, 7; over 60 and under 70, 13; over 70 and under 80, 4; over 80 and under 90, 2; unknown, 10. Total, 275.

NATIVITIES.

| | | | | | |
|---|---|---|---|---|---|
| Chicago, | 124 | Canada, | 7 | Switzerland, | 1 |
| Other States, | 47 | Ireland, | 26 | Holland, | 2 |
| England, | 4 | Scotland, | 3 | Unknown, | 13 |
| Germany, | 40 | Sweden, | 3 | | |
| Denmark, | 7 | Norway, | 4 | Total | 275 |

DIVISIONS.

| | | | | | | | |
|---|---|---|---|---|---|---|---|
| North, | 78 | South, | 78 | West, | 114 | Unknown, | 5 |
| Total, | | | | | 275 | | |

Surgical Instruments. We have received a very complete illustrated catalogue of surgical instruments of Geo. Tiemann & Co.'s manufacture, issued by their agents, Messrs. Bliss & Sharp, 144 Lake Street, who will furnish them to the profession on application.

Obituary Record.—Died, at Tunbridge Wells, on February 11, 1866, W. T. Brande, Esq., F.R.S., aged 81. Dr. B. early in life devoted himself to chemical studies, and was for some time assistant and afterwards the successor of Sir Humphrey Davy, as Professor of Chemistry to the Royal Institution of Great Britain. He was a voluminous writer.

Mortality for the Month of July.—Below will be found the mortality report for last month, as prepared by health-officer Bridges. It will be noticed that there is a great increase in the number of deaths not only over last month, but over the corresponding month last year; the number of deaths being much greater than in any month heretofore, either this year or during any previous year, except in that year when cholera made such ravages among our population. The great mortality last month was undoubtedly owing to the excessively hot weather, united to the carelessness of the people in matters of diet and to uncleanliness, a fruitful source, in itself, of many of the evils to which humanity is heir, in the summer season especially. The table below will show that the greater number of deaths have been from diseases more peculiarly belonging to summer. Of course, as the city has increased in the number of its population, even during the last twelve months, some allowance must be made for an extra large number of deaths, naturally resulting from that cause. The number of deaths for

July, 1865, was 425, and for the month just ended, 706, an increase of over 60 per cent. over the former month.

CAUSES OF DEATH.

| | | | |
|---|---|---|---|
| Accidents, | 14 | Fever, not stated, | 1 |
| Abortion, | 1 | Gravel, | 2 |
| Cancer, | 3 | Hydrocephelas, | 6 |
| Childbed, | 2 | Inflammation of Brain, | 15 |
| Cholera-Morbus, | 10 | Inflammation of Bowels, | 11 |
| Cholera Infantum, | 35 | Inflammation of Lungs, | 12 |
| Congestion of Brain, | 7 | Intemperance, | 3 |
| Congestion of Lungs, | 4 | Killed, | 3 |
| Consumption, | 28 | Measles, | 67 |
| Convulsions, | 59 | Marasmus, | 1 |
| Croup, | 15 | Old Age, | 13 |
| Delirium Tremens, | 2 | Paralysis, | 1 |
| Decline, | 3 | Rheumatism, | 2 |
| Diarrhœa, | 21 | Small-Pox, | 2 |
| Diarrhœa, Chronic, | 2 | Stillborn, | 12 |
| Diphtheria, | 6 | Summer Complaint, | 159 |
| Disease of Heart, | 10 | Suicide, | 2 |
| Disease of Liver, | 5 | Sunstroke, | 2 |
| Disease of Throat, | 1 | Teething, | 30 |
| Dropsy, | 3 | Tumor, | 7 |
| Drowned, | 5 | Typhoid Pneumonia, | 2 |
| Dysentery, | 29 | Whooping-Cough, | 15 |
| Fever, Remittent, | 1 | Worms, | 1 |
| Fever, Childbed, | 1 | Unknown, | 42 |
| Fever, Scarlet, | 14 | | |
| Fever, Typhus, | 1 | Total, | 706 |
| Fever, Typhoid, | 18 | | |

Ages of the Deceased.—Under 5 years, 518; over 5 and under 10 years, 33; over 10 and under 20, 13; over 20 and under 30, 35; over 30 and under 40, 45; over 40 and under 50, 22; over 50 and under 60, 12; over 60 and under 70, 9; over 70 and under 80, 9; over 80 and under 90, 1; over 90 and 100, 1; unknown, 8. Total, 706.

NATIVITIES.

| | | | | | |
|---|---|---|---|---|---|
| Chicago, | 474 | France, | 1 | Mexico, | 1 |
| Other States, | 72 | Holland, | 3 | Switzerland, | 2 |
| Canada, | 3 | Ireland, | 44 | Sweden, | 6 |
| Denmark, | 2 | Norway, | 13 | Unknown, | 9 |
| England, | 6 | Bohemia, | 1 | | |
| Germany, | 62 | Scotland, | 3 | Total, | 706 |

DIVISIONS.

North, 190 | South, 196 | West, 315 | Country, 5
Total, 706

Deodorizing Properties of Ground Coffee.—Dr. Barbier affirms that ground coffee possesses some remarkable properties as a disinfectant. In several cases where he had to make *post mortem* examinations of bodies under *very disagreeable* circumstances, he found that a handful of coffee strewn over the body and about the room quite overcame any bad odor.—*Lancet.*

